# MiT family translocation renal cell carcinoma with retroperitoneal metastasis in childhood: a case report

**DOI:** 10.3389/fped.2023.1141223

**Published:** 2023-07-17

**Authors:** Kaihang Yang, Yuhao Ma, Shuyang Dai, Rui Dong

**Affiliations:** Department of Pediatric Surgery, Shanghai Key Laboratory of Birth Defect, Children's Hospital of Fudan University, Shanghai, China

**Keywords:** MiT family translocation renal cell carcinoma, Xp11.2 translocation, TFE3, partial nephrectomy (PN), retroperitoneal metastasis

## Abstract

RCC accounts for only 0.1%–0.3% of all kidney tumors and 2%–6% of malignant kidney tumors in children. Accounting for approximately one-third of the total number of cases in children and adolescents with RCC, Xp11.2 tRCC is the most common subtype of the MiT family translocation renal cell carcinoma, which is a group of rare childhood and adult tumors, characterized by recurrent gene rearrangements of TFE3. Here we report a rare case of a 6-year-old male patient with MiT family translocation renal cell carcinoma (MiTF tRCC) where the patient developed retroperitoneal metastasis. The patient underwent partial nephrectomy (PN), radical nephrectomy (RN), abdominal lymph node resection, and intestinal adhesion lysis. Microscopically, we detected focal and nest clump-shaped clusters of tumor cells whose cytoplasm was bright and clear. Immunohistochemistry (IHC) showed tumor cells diffusely expressed TFE3, and fluorescence *in situ* hybridization (FISH) demonstrated disruption of the TFE3 locus, confirming the diagnosis of Xp11.2 tRCC, the most common subtype of MiTF tRCC. Eventually, the patient obtained a good therapeutic result. This case can provide a good reference and guidance for pediatric urologists and oncologists to recognize and diagnose rare renal cell carcinoma in children.

## Introduction

MiT (microphthalmia transcription factor) family translocation renal cell carcinoma (MiTF tRCC) is a group of rare childhood and adult tumors that includes two subtypes, Xp11.2 translocation RCC and *t*(6;11) RCC, which are characterized by recurrent gene rearrangements of TFE3 and transcription factor EB (TFEB) loci respectively. The MiT subfamily of transcription factors includes MiTF, TFE3, TFEB, and TFEC (1). Both TFE3 and TFEB belong to the MiT family genetically, and there are clinical, morphologic, and immunohistochemical similarities between the two subtypes of RCC; these tumors were therefore grouped as belonging to the MiT family translocation RCC (MiT-RCC) by the World Health Organization in 2016 (2). As the most common subtype of the MiT family translocation renal cell carcinoma and accounting for nearly 50% of RCC in childhood, the Xp11.2 tRCC harbors gene fusions involving TFE3 with one of multiple reported genes, including ASPSCR1 (ASPL), PRCC, NONO, SFPQ, CLTC, PARP14, LUC7L3, DVL2, KHSRP, and RBM10, which was officially recognized in the 2004 WHO renal tumor classification for the first time. Compared with other RCC generally, the Xp11.2 tRCC has a similar clinical presentation, and surgery is regarded as the most common and effective therapeutic method. Pediatric patients often have a better prognosis than adults (3).

Here we report a rare case of a 6-year-old male patient with MiTF tRCC who developed retroperitoneal metastasis.

## Case report

**Figure 1:**
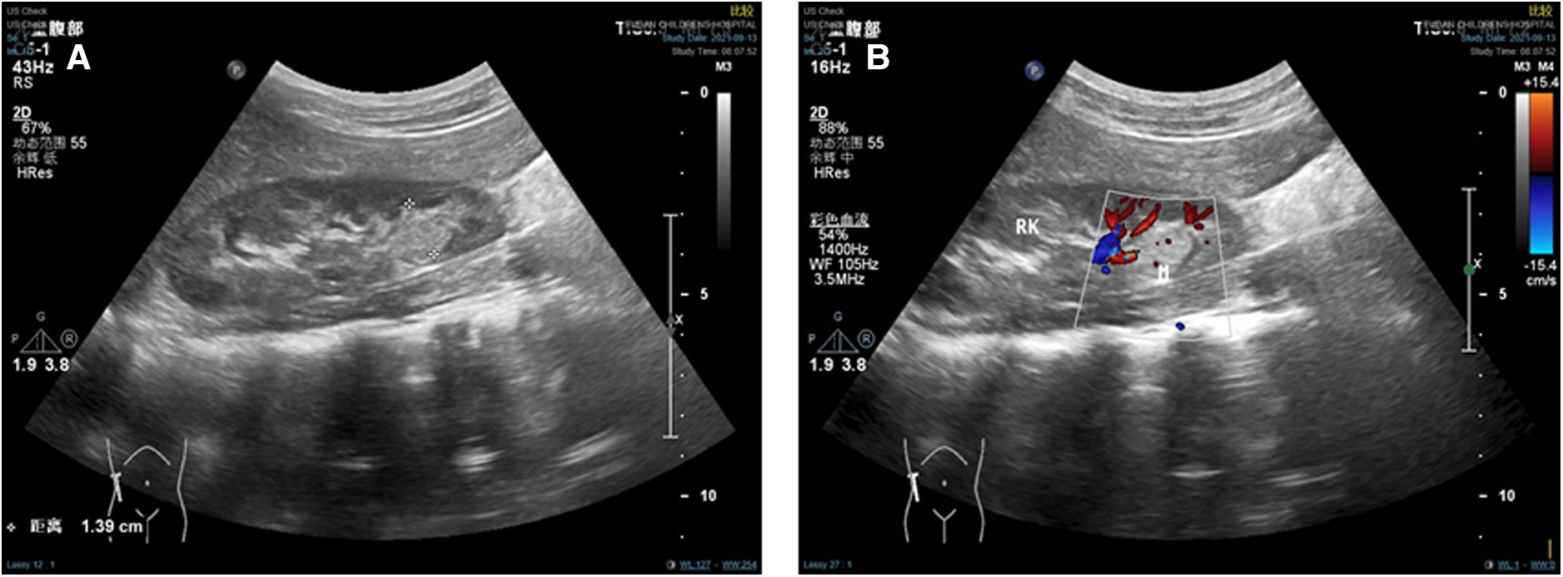
Preoperative ultrasound results. (**A**) Ultrasound showed a moderate echogenic area at the dorsal side of the lower pole of the right kidney. (**B**) Ultrasound showed blood flow signal inside the echogenicity.

A 6-year-old boy suffered from abdominal pain without apparent cause 2 months before the operation, the course of the pain lasted for 3 days, and the pain went away gradually. After questioning the medical history, the patient reported gross hematuria. At the same time, an abdominal mass was found upon physical examination. On 8 July 2021, the patient underwent exploratory laparotomy and retroperitoneal mass resection at a local tertiary general hospital; the postoperative pathological biopsy examination of lymph nodes revealed that the tumor cells in the lymph tissue were distributed in nests and papillary, and the cytoplasm was clear, suggesting that renal cancer with retroperitoneal lymph node metastasis. The results of the pathological biopsy above had been confirmed by pathological consultation in our hospital. On 10 September 2021, the blood routine test results were as follows. CRP: 21 mg/L↑; Hb: 136 g/L; LY%: 50.7%↑; GR%: 36.4%↓; red blood cell count (RBC): 4.87 × 10^12^/L; white blood cell count (WBC): 5.15 × 10^9^/L. On 13 September 2021, the ultrasound results showed that the size of the left kidney was 82.7 mm × 29.5 mm × 30.3 mm and the left kidney had no obvious abnormal internal echogenicity, clear structure, normal morphology, smooth envelope, no widening of the renal collecting system, and normal blood supply. The size of the right kidney was 88.7 mm × 24.6 mm × 31.6 mm, and the structure of the right kidney was unclear. There was a moderate echogenic area at the dorsal side of the lower pole of the right kidney near the renal envelope, and the size of the echogenic area was 22.8 mm × 13.9 mm × 17.8 mm. Significantly, the internal echogenicity of the area was heterogeneous with unclear borders and irregular morphology, and there was a blood flow signal inside the echogenicity, suggesting parenchymal occupancy at the dorsal side of the lower pole of the right kidney near the envelope (Figures 1A,B). Preoperative ultrasound results indicated a retroperitoneal tumor.

**Figure 2:**
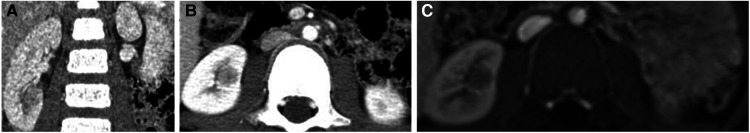
Upper abdominal computed tomography showed a slightly dense nodular occupancy in the lower pole of the right kidney. (**A**) Coronal plane. (**B**) Transverse plane. (**C**) MRI showed a circular nodal shadow on the lower pole of the right kidney.

The result of the CT scan showed a circular iso-density shadow measuring 16.3 mm × 11.9 mm × 9.4 mm on the lower pole of the right kidney on 13 September 2021 (Figures 2A,B). Additionally, MRI result also showed a circular nodal shadow measuring 10 mm × 9 mm on the lower pole of the right kidney on 14 September 2021 (Figure 2C). The pediatric patient did not have pulmonary or hepatic metastasis, so imaging of the lungs and liver was not required. To further evaluate the perfusion and function of the kidneys, a DTPA examination was performed on 15 September 2021. The unstandardized GFR of both kidneys was 103.9 ml/min (100 ± 10 ml/min), and the GFR of the left kidney was 51.7 ml/min, while the GFR of the right kidney was 52.2 ml/min. The DTPA examination results showed that the renal function and perfusion with the excretion of the left kidney were slightly delayed compared to the right kidney. Eventually, the boy was treated with partial nephrectomy (PN) and radical nephrectomy (RN) mainly at the Children's Hospital of Fudan University on 16 September 2021 and was diagnosed with malignant renal cell carcinoma with retroperitoneal metastasis, which belongs to the MiT family translocation renal cell carcinoma according to the postoperative immunohistochemistry (IHC: 23 September 2021) and fluorescence *in situ* hybridization (FISH: 18 October 2021) result.

## Results

**Figure 3:**
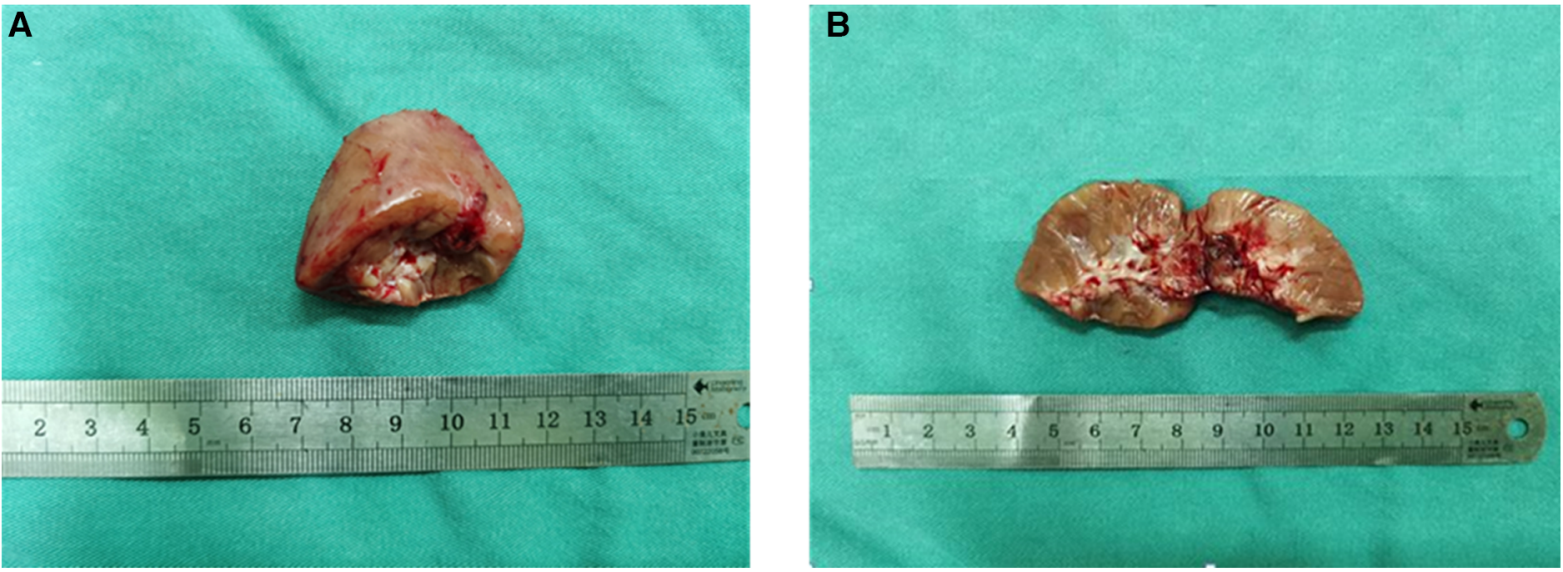
(**A**) The kidney tissue specimen cut from the lower pole of the right kidney during the operation is gray-red in color and approximately 3.5 cm × 3 cm × 1.5 cm in size. (**B**) The cut surface of the kidney tissue is gray-red, and the central part is gray-white and gray-brown, with an area of about 1 cm × 1 cm and soft texture.

**Figure 4:**
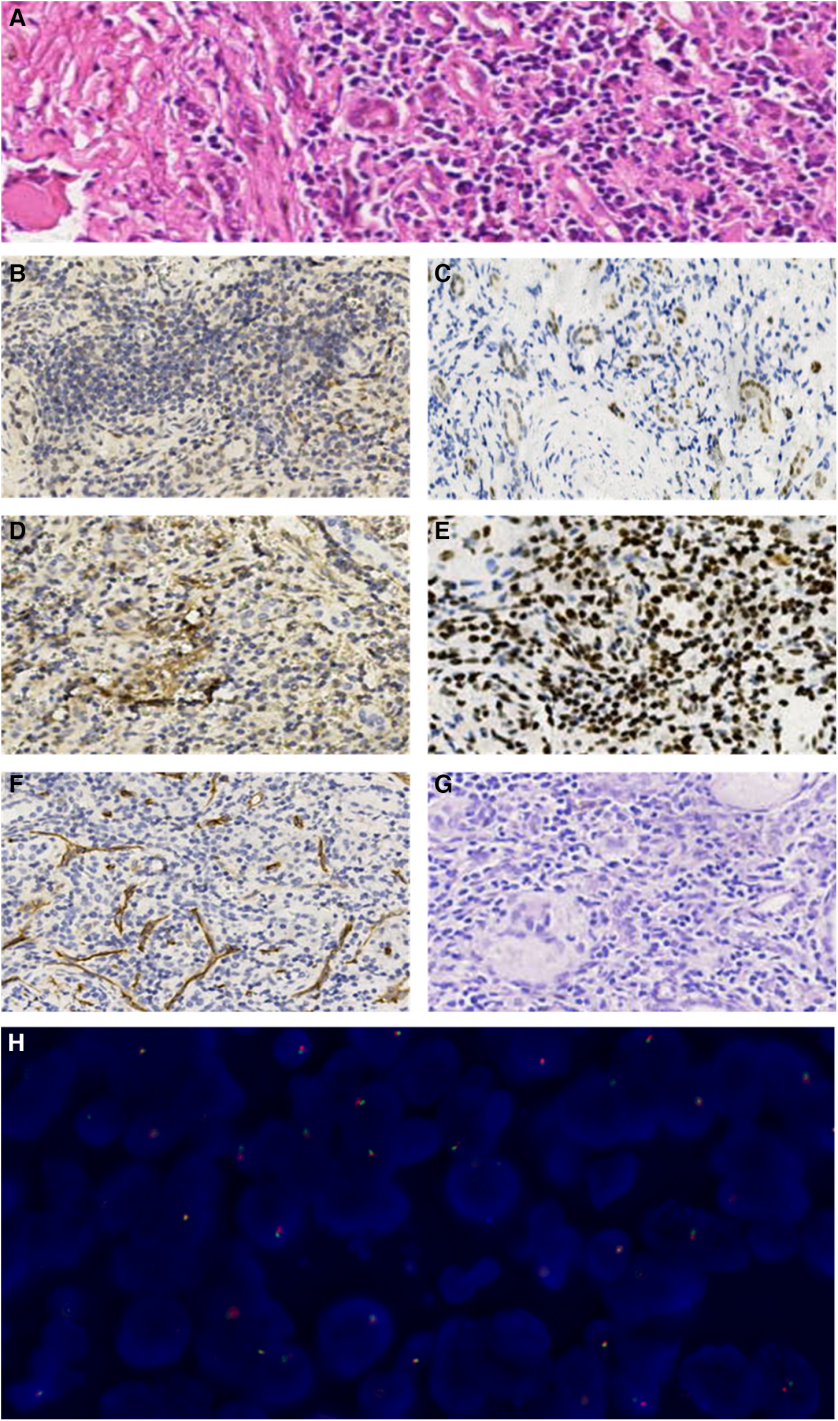
(**A**) The microscopic examination revealed focal and nest clump-shaped clusters of tumor cells whose cytoplasm was clear and bright, whose nucleolus was obviously enlarged, and where scattered gravel bodies were visible (HE staining, ×20). (**B–G**). Immunohistochemical manifestations of the tumor cells (×10). (**B)** TFE3(+). (**C**) PAX8(+). (**D**) Vimentin(+). (**E**) INI-1(+). (**F**) CD34(−). (**G**) D-PAS(−). (**H**) The FISH result showed that more than one-fifth of the tumor cells showed separation of green and red signals with a positive propensity for disruption and rearrangement of TFE3.

Three complementary radiodiagnosis examinations (CT, MRI, and DTPA), Immuno- -histochemistry (IHC), fluorescence *in situ* hybridization (FISH), and a review of clinical medical records contributed to the patient's final diagnosis. The boy underwent partial nephrectomy (PN), radical nephrectomy (RN), abdominal lymph node resection, and intestinal adhesion lysis at the Children's Hospital of Fudan University on 16 September 2021 after intraoperative freeing of the right kidney a mass was seen in the lower pole of the right kidney. The surgical specimen demonstrated a tumor measuring 2.5 cm × 2 cm × 2 cm on the lower pole of the right kidney (Figures 3A,B). On 23 September 2021, the microscopy result of the pathological tissue showed focal and nest clump-shaped clusters of tumor cells with clear and bright cytoplasm; some of the nuclei were significantly enlarged, and scattered gravel bodies were visible (Figure 4A). The pathological biopsy of the patient had the classic histological features of MiTF tRCC, as assessed and confirmed by first-time pathologist Feng Tian and follow-up pathologist Yangyang Ma of the Department of Pathology, Children's Hospital of Fudan University. Immunohistochemistry (IHC) showed the tumor cells were positive for TFE3 (Figure 4B), PAX8 (Figure 4C), Vimentin (Figure 4D), INI-1 (Figure 4E), CD10, CK, ki-67(3%+), and PAS and were negative for CD34 (Figure 4F), D-PAS (Figure 4G), PAX2, and PHOX2B.

On 18 October 2021, a genetic diagnosis was performed. The TFE3 (Xp11.2) gene locus was detected by using the TFE3 gene dual-color separation probe. The fluorescence *in situ* hybridization (FISH) result demonstrated that more than one-fifth of the tumor cells were detected to have red and green signals separation and the number of cells counted was 200 with the positive propensity for disruption and rearrangement of TFE3 (Figure 4H). The postoperative pathology test reports above suggest that the boy was diagnosed with Xp11.2 translocation/TFE3 fusion-associated RCC, the most common subtype of the MiTF tRCC. The boy and his parents were satisfied with the result of the surgical treatment he received, and the boy was in good condition after the operation eventually. The child was given oral targeted drugs postoperatively, sunitinib, for 4 weeks with a 2-week discontinuation and intermittent treatment intervals based on the patient's safety and tolerability. At 18 months of follow-up, the child did not show any significant tumor recurrence or metastasis, and the current physiological indexes were normal.

## Discussion

In children and adolescents, Wilms tumor (WT) is the most common malignant renal tumor, while RCC accounts for only 0.1%–0.3% of all renal tumors and 2%–6% of malignant renal tumors (4). Compared with WT in children, RCC has an older onset age and is more common in boys over 5 years old. The age and sex characteristics of the pediatric patient were consistent with the typical cases reported in the literature. Xp11.2 translocation/TFE3 gene fusion-associated RCC is characterized by translocations at different loci on the Xp11.2 chromosome, resulting in a fusion between TFE3 and its partner gene. In previously reported literature, ASPSCR1/ASPL-TFE3 and PRCC-TFE3 were the most frequently detected partner genes, followed by SFPQ/PSF-TFE3, NONO-TFE3, CLTC-TFE3, while other partner genes such as RMB10-TFE3, DVL2-TFE3, etc. are rarely reported (5). Most patients with Xpl1.2 RCC have no clinical manifestations, and only a small number of patients may present with gross hematuria, lower back pain, and abdominal mass, which are defined as the renal cancer triad. Moreover, most of the symptomatic patients present with only one of the three symptoms, and gross hematuria is the most common clinical manifestation, while renal cancer triad is rare. Therefore, the symptoms of Xpl1.2 RCC are similar to those of other common types of renal tumors and have no specificity (6). The pediatric patient in this case presented with a rare renal cancer triad.

Abdominal ultrasonography is the easiest and most commonly used method for the primary screening of renal tumors, and it can be used as a daily inspection method for postoperative follow-up. Abdominal CT examination has high diagnostic sensitivity and specificity and can be used for qualitative diagnosis of most renal tumors. Meanwhile, abdominal CT examination is the most commonly used examination method for preoperative diagnosis and postoperative follow-up of RCC in children. MRI examination is non-radioactive and suitable for patients who are allergic to contrast agents, which is also a common examination method for preoperative diagnosis and postoperative follow-up of RCC in children. Based on three complementary diagnostic imaging findings [CT, MRI, and Diethylenetriamine pentaacetate dynamics in magnetic resonance imaging examination (DTPA)], we still need differential diagnoses to confirm the clinical diagnosis. Wilms tumor, also known as nephroblastoma, is the most common kidney cancer in children. It is also the most common pediatric abdominal cancer and the fourth most common pediatric cancer. Wilms tumor is typically found in children younger than 5 years old and most often affects children ages 3–4, but it can still affect older children and even adults. The tumor is named after Dr. Max Wilms, a German physician who first described it in 1899. The prognosis varies according to tumor stage and histology. Favorable histology has survival rates of 99% to 86%, while the survival rate of people with unfavorable histology ranges from 84% to 38% depending on the stage. Over the years, progress in the diagnosis and treatment of Wilms tumor has greatly improved the prognosis for children with WT (7). WT has a high metastasis rate to the lung and liver through blood. The CT result of WT shows a solid mass with a pseudocapsule inside the kidney, which compresses the renal parenchyma and interstitium (8). Although the pathological test is regarded widely as the gold standard for confirming WT, the radiologic test still plays a vital role in the WT diagnosis because WT lacks specific tumor markers (9). Retroperitoneal teratoma is a primary tumor originating from primitive germ layer tissue, which has a relatively long course. Most retroperitoneal teratomas are benign, but a small number of malignant tumors usually metastasize to the lungs. The CT result of retroperitoneal teratoma often shows the following details: (1) there are some images of fat, calcification, or ossification in the liquid low-density area; (2) CT shows enhancement of low to moderate density while MRI presents short T1 and slightly longer T2; (3) indirect signs conclude compression and displacement of tissues and organs surrounding the tumor (10).

The most typical pathological morphology of Xp11.2 translocation RCC is a papillary structure composed of clear cells with calcified nodules (HE staining). The morphology of different fusion gene subtypes of RCC is not the same. For example, the tumor cells of the PRCC-TFE3 type have less cytoplasm, are arranged in the shape of nests, papillary, and glandular tubes, and gravel bodies are less common. In contrast, the tumor cells of ASPL-TFE3 type are large and abundant in cytoplasm, with scattered cell borders, prominent nucleoli, and a large number of gravel bodies (11). In this case, the microscopic examination revealed focal and nest clump-shaped clusters of tumor cells whose cytoplasm was clear and bright, whose nucleolus was obviously enlarged, and where scattered gravel bodies were visible, which was consistent with the morphological appearance of ASPL-TFE3 type reported in the literature. Therefore, it was considered that the TFE3 partner gene for this patient's tumor might be ASPL-TFE3. The expression of nuclear TFE3 protein is a characteristic manifestation of Xpll.2 translocation RCC (IHC staining). The sensitivity and specificity of TFE3 protein expression in diagnosing MiTF RCC are 97.5% and 99.6% respectively (12), so the results of IHC staining of TFE3 are the main basis for the diagnosis of Xpll.2 RCC. In addition, compared with most RCC, MiTF RCC usually shows low expression of epithelial markers, high expression of nephrogenic marker PAX8, and generally no expression of CK7, CD117, and carbonic anhydrase IX. TFE3 tRCC often expresses CD10 and AMACR and occasionally expresses melanocyte markers such as Melan-A, HMB-45, and cathepsin K (13). Jian Wu et al., collected and analyzed the clinicopathological data of 10 patients with Xpll.2 RCC. Immunohistochemistry of 10 patients with Xpll.2 RCC showed that all 10 tumor tissues expressed TFE3, Melan-A, p504s and CK and also expressed vimentin, CD10, RCC, PAX8, and EMA to varying degrees, but did not express CK7 and CD117 (14). According to Cajaiba M M et al., approximately 16%–24% of pediatric RCC cannot be classified into specific subtypes. In their review of 168 pediatric RCC prospectively registered on Children's Oncology Group AREN03B2 protocol, 6 cases (3.5%) showed expression of ALK and TFE3 and retention of INI1 (BAF47), and ALK rearrangements in all cases were identified, so they proposed that these neoplasms belonged to a distinct subgroup of childhood RCC (ALK-rearranged RCC). Some scholars believe that the retention of nuclear INI-1 (BAF47) is a differential feature of ALK- rearranged RCC and other renal medullary carcinoma (RMC) (15). In this case, the IHC results of Xp11.2 translocation/TFE3 gene fusion-associated RCC were TFE3(+), PAX8(+), Vimentin(+), and INI-1 (BAF47) (+), which were consistent with the literature reports. Genetic detection of Xp11.2 translocation is the gold standard for the diagnosis of Xp11.2 translocation/TFE3 fusion-associated RCC (16), but this technique is relatively complicated and the detection cost is high, so it has not been widely carried out yet.

Regardless of the pathological subtype of RCC in children, surgery is the main treatment, and children with localized RCC can obtain a favorable prognosis just through surgery. As a classic surgical procedure for RCC in children, radical nephrectomy (RN) resects the affected kidney, perirenal fascia, perirenal fat, ipsilateral adrenal gland, lymph nodes from the crus of the diaphragm to the bifurcation of the abdominal aorta, and above the bifurcation of the iliac vessels ureter, but the latest recommendations no longer recommend routine adrenalectomy and regional lymph node dissection. Partial nephrectomy (PN), also known as nephron-sparing surgery (NSS), has been widely performed in pediatric and adult patients with RCC. For adult patients, PN may be considered for renal tumors with a diameter <7 cm, clear borders, and a location at the edge of the kidney. At present, there are few clinical reports of PN in children with RCC. However, the biological behavior of translocation RCC in childhood is inert, indications for PN in children with RCC should be very strict. Chao Liu et al., performed PN on 11 cases of RCC in children, and the results showed the diameter of renal tumors was 2.2–6.9 cm with an average of 3.3 cm. Therefore, it is considered safe and feasible to perform PN for translocation RCC with a diameter <7 cm in children (17). Qiuhong Ma et al., treated 3 cases of tRCC and retrieved relevant literature on RCC associated with Xpll.2 translocation/TFE3 gene fusion in children before November 2019 and found that 10 cases had retroperitoneal metastasis and para-aortic lymph node metastasis, 4 cases had vena cava metastasis, and 3 cases had distant metastasis. She believes that RCC associated with Xpll.2 translocation/TFE3 gene fusion in children is prone to lymph node metastasis. Lymph node biopsy is recommended after lymph node dissection when the patient presents with enlarged lymph node metastases (18). In summary, surgical resection is the first choice for the treatment of translocation RCC in children, and pediatric patients with or without regional lymph node metastasis should receive radical nephrectomy or partial nephrectomy (19). The patient in this case was 6 years old, and the tumor size was 2.5 cm × 2 cm × 2 cm with retroperitoneal metastasis. Therefore, partial nephrectomy (PN), radical nephrectomy (RN), and abdominal lymph node resection were performed. The patient underwent exploratory laparotomy and retroperitoneal mass resection at another hospital on 8 July 2021 before the diagnosis of MiT family translocation renal cell carcinoma with retroperitoneal metastasis was confirmed. Postoperative intestinal adhesion occurred, and perirenal exudation and adhesion were observed in our surgical field of view, so intestinal adhesion lysis was performed during the operation.

Targeted drugs have a certain therapeutic effect on translocation RCC with metastasis, but it is still necessary to be cautious in using targeted therapy in children with tRCC. Sunitinib is a multi-targeted tyrosinase inhibitor that inhibits multiple growth factor receptors. It has been used in adult patients, but its efficacy in pediatric patients remains to be seen. Sudour-Bonnange, H. et al. (2014) reported a case of Xp11.2 translocation RCC with skin metastasis in a 15-year-old patient. Their study showed that targeted agents such as sorafenib and sunitinib can prolong the lifetime of patients diagnosed with Xp11.2 translocation RCC with lymph node metastasis or distant metastasis without progression (20). However, there is no literature to support a great survival benefit with systemic chemotherapy or radiotherapy alone.

In summary, to our knowledge, renal cell carcinoma in children is less common than malignant nephroblastoma. There are no specific clinical manifestations, imaging preoperative diagnosis is difficult, treatment experience is scarce, and there are even fewer published articles on MiT family translocation renal cell carcinoma with retroperitoneal metastasis in pediatric patients. This is a rare case of Xp11.2 tRCC in a child with retroperitoneal metastasis. This article analyzes a single case of renal cell carcinoma from a single center in the field of pediatrics, describing and analyzing the clinical and imaging manifestations of Xp11.2 translocation/TFE3 gene fusion-associated RCC, which predominates in children with renal cell carcinoma, as well as the pathological findings of resected tumor specimens. Treatment was mainly based on radical nephrectomy, and a good therapeutic result was obtained in this case. There is a good reference value and guidance for pediatric urologists and oncologists to recognize and diagnose renal cell carcinoma in children.

## Data Availability

The original contributions presented in the study are included in the article/Supplementary Material, further inquiries can be directed to the corresponding author.
