# Supplementary material for: MiT family translocation renal cell carcinoma with retroperitoneal metastasis in childhood: a case report
Source: Front Pediatr. 2023 Jul 17;11:1141223. doi: 10.3389/fped.2023.1141223 (PMC10388245; doi:10.3389/fped.2023.1141223)
Supplement: Supplementary file 1 [file Table1.docx]

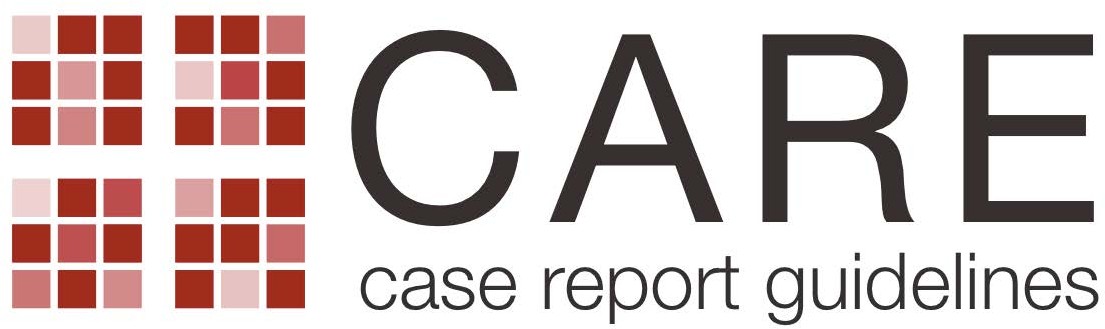
CARE Checklist of information to include when writing a case report
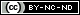


**Topic Item Checklist item description Reported on Line**

**Title 1** The diagnosis or intervention of primary focus followed by the words “case report” **p.1, line1-2**

**Key Words 2** 2 to 5 key words that identify diagnoses or interventions in this case report, including "case report"  **p.2, line16-17**

**Abstract**

**(no references)**

**3a** Introduction: What is unique about this case and what does it add to the scientific literature? …………………**p.2,line18-22,p.3,line1-13**

**3b** Main symptoms and/or important clinical findings **p.3, line15-22, p.4**

**3c** The main diagnoses, therapeutic interventions, and outcomes ………………………………………………………**p.5,line1-4, p.8,line3-7**

**3d** Conclusion—What is the main “take-away” lesson(s) from this case? **p.8, line8-18**

**Introduction 4** One or two paragraphs summarizing why this case is unique (**may include** reference**s**) ……………**p.2,line13-15,p.8,line8-18**

**Patient Information 5a** De-identified patient specific information **p.3, line15-16**

**5b** Primary concerns and symptoms of the patient **p.3, line15-19**

**5c** Medical, family, and psycho-social history including relevant genetic information **p.3, line15-22,p.4,p.8,line1-5**

**5d** Relevant past interventions with outcomes **p.3,line15-16**

**Clinical Findings**

**Timeline**

**Diagnostic Assessment**

**Therapeutic Intervention**

**Follow-up and Outcomes**

1. Describe significant physical examination (PE) and important clinical findings **p.3, line16-22,p.4**
2. Historical and current information from this episode of care organized as a timeline **p.5, line1-4**

**8a** Diagnostic testing (such as PE, laboratory testing, imaging, surveys). **p.3, line16-22, p.4**

**8b** Diagnostic challenges (such as access to testing, financial, or cultural) **Not applicable**

**8c** Diagnosis (including other diagnoses considered) ……………………………………………………………...**p.5, line2-4,p.8,line3-5**

**8d** Prognosis (such as staging in oncology) where applicable **p.8, line5-7**

**9a** Types of therapeutic intervention (such as pharmacologic, surgical, preventive, self-care) **p.6, line5-7**

**9b** Administration of therapeutic intervention (such as dosage, strength, duration) **p.6, line5-7**

**9c** Changes in therapeutic intervention (with rationale) **Not applicable**

**10a** Clinician and patient-assessed outcomes (if available) **p.8, line5-7**

**10b** Important follow-up diagnostic and other test results …………………………………………………………………..**p.6,line9-14,p.8,line1-7**

**10c** Intervention adherence and tolerability (How was this assessed?) **Not applicable**

**10d** Adverse and unanticipated events **Not applicable**

**Discussion 11a** A scientific discussion of the strengths AND limitations associated with this case report ………………………. **p.5,line5-17,p.6,p.8,line8-18**

**11b** Discussion of the relevant medical literature **with references** **p.5,line5-17,p.6,line1-2**

**11c** The scientific rationale for any conclusions (including assessment of possible causes) .. **p.5,line5-17,p.6,line1-2**

**11d** The primary “take-away” lessons of this case report (without references) in a one paragraph conclusion ……**p.8,line8-18**

**Patient Perspective 12** The patient should share their perspective in one to two paragraphs on the treatment(s) they received **Not applicable**

**Informed Consent 13** Did the patient give informed consent? Please provide if requested . . . . . . . . . . . . . . . . . . . . . . . . . . . . . . . . . . . . . . **Yes No**
